# Indirect and Improper Advertising

**Published:** 1887-11

**Authors:** 


Indirect and Improper Advertising.—The following remarks in the form of an editorial in the *Journal of the American Medical Association*, is applicable to dental as well as medical advertisements:

"We frequently receive from some of those who have advertisements in the advertising pages of *The Journal*, printed slips ingeniously calling attention to the value or peculiar quality of one or more of the articles they advertise, couched in such phraseology as to appear to have been written by the editor of *The Journal*, accompanied by a request to give such slips a place in the columns of *The Journal* for reading matter. We have uniformly refused to use such slips, and for the following reasons: 1. In making a contract with an advertising patron for a given space in the advertising columns and for a certain sum, there is no condition either expressed or implied that he shall have any additional space in any other columns not devoted to advertisements. 2. If we should accept such slips and place them in our reading columns as though they were expressions of our own opinion concerning this or that article, when they were actually only the interested expressions of the manufacturers or advertisers themselves, we would be practicing a direct fraud upon our readers. This reason alone, is abundantly sufficient to deter us from yielding to any such use of our columns. Not a few enthusiastic manufacturers of medicines, formulas, foods, etc., appear to think we should actually test by clinical use every new thing, and every new combination of old things, they choose to send us, and express either by certificate or through the columns of *The Journal*, our most gratifying approval. It seems never to have entered into the thoughts of such parties, that such a task would require us to devote *all our time* to that work, and make *all* our patients subjects for *experimental* dosing, leaving us neither time to edit *The Journal* or even to tabulate the results of our heterogenous experiments on suffering humanity.

It is proper for physicians to use cautiously and judiciously such new remedies as may reasonably be presumed to possess valuable properties in the treatment of disease. But to obtain reliable results, such use must be extended through a large number of cases, the correct diagnosis of which has been assured; and only an actual clinical record of such cases becomes appropriate and desirable material for the reading columns of medical journals."

---

